# Objectively measured physical activity in four-year-old British children: a cross-sectional analysis of activity patterns segmented across the day

**DOI:** 10.1186/1479-5868-11-1

**Published:** 2014-01-09

**Authors:** Kathryn R Hesketh, Alison M McMinn, Ulf Ekelund, Stephen J Sharp, Paul J Collings, Nicholas C Harvey, Keith M Godfrey, Hazel M Inskip, Cyrus Cooper, Esther MF van Sluijs

**Affiliations:** 1 UKCRC Centre of Excellence for Diet and Activity Research (CEDAR), Box 296, Institute of Public Health, University of Cambridge, Cambridge, UK; 2 MRC Epidemiology Unit, Institute of Metabolic Science, Box 285, Addenbrooke's Hospital, Cambridge, UK; 3 Department of Sport Medicine, Norwegian School of Sport Sciences, Oslo, Norway; 4 MRC Lifecourse Epidemiology Unit, University of Southampton, Southampton General Hospital, Southampton, UK; 5 NIHR Southampton Biomedical Research Centre, University of Southampton and University Hospital Southampton NHS Foundation Trust, Southampton, UK

## Abstract

**Background:**

Little is known about preschool-aged children’s levels of physical activity (PA) over the course of the day. Using time-stamped data, we describe the levels and patterns of PA in a population-based sample of four-year-old British children.

**Methods:**

Within the Southampton Women’s Survey the PA levels of 593 4-year-old children (51% female) were measured using (Actiheart) accelerometry for up to 7 days. Three outcome measures: minutes spent sedentary (<20 cpm); in light (LPA: ≥20 – 399 cpm) and in moderate-to-vigorous activity (MVPA: ≥400 cpm) were derived. Average daily activity levels were calculated and then segmented across the day (morning, afternoon and evening). MVPA was log-transformed. Two-level random intercept models were used to analyse associations between activity level and temporal and demographic factors.

**Results:**

Children were active for 67% (mean 568.5 SD 79.5 minutes) of their daily registered time on average, with 88% of active time spent in LPA. All children met current UK guidelines of 180 minutes of daily activity. There were no differences in children’s average daily levels of sedentary activity and LPA by temporal and demographic factors: differences did emerge when activity was segmented across the day. Sex differences were largest in the morning, with girls being more sedentary, spending fewer minutes in LPA and 18% less time in MVPA than boys. Children were more sedentary and less active (LPA and MVPA) in the morning if they attended childcare full-time compared to part-time, and on weekend mornings compared to weekdays. The reverse was true for weekend afternoons and evenings. Children with more educated mothers were less active in the evenings. Children were less sedentary and did more MVPA on summer evenings compared to winter evenings.

**Conclusions:**

Preschool-aged children meet current physical activity guidelines, but with the majority of their active time spent in LPA, investigation of the importance of activity intensity in younger children is needed. Activity levels over the day differed by demographic and temporal factors, highlighting the need to consider temporality in future interventions. Increasing girls’ morning activity and providing opportunities for daytime activity in winter months may be worthwhile.

## Introduction

Physical activity plays an important role in children’s physical and mental wellbeing [1,2], with particular benefits for preschool children’s (3-5 years) social, gross motor and development skills [3,4]. Although the long-term benefits of being physically active from a very young age have yet to be confirmed, emerging evidence suggests that active children under 5 have better health and cognitive outcomes compared to less active peers, with positive habit formation early in life likely to be beneficial [5]. Establishing a high baseline level of physical activity when young could be especially important given that activity levels are known to decrease from late childhood, through adolescence into adulthood [6,7].

Whilst there is a perception that young children are constantly active [8,9], prevalence estimates suggest that this may not be the case. A review in 2010 found that preschool children accrue only limited amounts of physical activity during the preschool day, are sedentary for large amounts of time, and fail to meet the then recommended activity guidelines (>60 minutes MVPA) [10]. These guidelines, from National Association for Sport and Physical Education (NASPE), advocated 60 minutes of structured physical activity, with at least 60 minutes and up to several hours of further unstructured play each day for the under-5 s [11]. Yet when adherence to these recommendations was assessed across 39 studies, only 54% of children met these guidelines [12].

More recently, new physical activity guidelines for preschool-aged children have been published in Canada [13], Australia [14], the UK [15], and the USA [16]. Broadly similar, they recommend preschool-aged children should be active for 180 minutes daily. Importantly, these guidelines do not specify activity intensity, instead focusing on maximising activity throughout the day [13-15]. Recommendations for sedentary behaviour in this age group also vary internationally, with UK recommendations stating that the time children spend sedentary or restrained when not asleep should be limited [15]. Other guidelines advocate limiting screen time to 1 hour or less per day (Australia [14]) or restricting sedentary time to less than 1 hour at a time (US [16]). Using these new guidelines, published evidence from Australia [17] suggests young children are active for around 130 minutes on average each day, equating to roughly 16% of measured time. The amount of moderate-to-vigorous physical activity (MVPA) children engaged was approximately 4.8% [17].

Most studies investigating activity levels in preschool children have focused on average activity levels. However, there is some suggestion that activity patterns differ over the course of the day and week [18]. Understanding differences in activity levels over the course of the day allows focussed intervention efforts to be developed, taking into account when children may be less active and therefore predisposed to efforts to increase activity. This paper aims to describe levels and patterns of activity in a population-based sample of 4-year-old British children. In addition to assessing children’s average activity levels, we were also able to explore how activity patterns change across the day, segmenting activity using hourly time-stamped data. These novel analyses therefore allow us to determine how children’s differing intensities of activity change throughout the day and how temporal and demographic factors influence this activity.

## Methods

### Study design and setting

The Southampton Women’s Survey (SWS) is a population-based prospective cohort study based in Southampton, UK [19]. The study was designed to assess maternal diet and lifestyle before and during pregnancy, with 12,583 women recruited from General Practices in the Southampton area between 1998 and 2002. Subsequent live births (n = 3,159) were followed-up at specific ages to examine how children’s pre-natal development interacts with their postnatal growth, and how both may affect their risk factors for a range of future chronic diseases [19]. Parents of all participating children gave full and informed written consent. Ethical approval for the study was granted by the Southampton and South West Hampshire Local Research Ethics Committee.

### Participants

This study uses data collected between March 2006 and June 2009 from a sample of 4-year-old children who were invited to participate in a sub-study (n = 1,065). Children were invited to attend a hospital visit to undergo body composition analysis and physical activity measurement in order to investigate the association between bone health, obesity and physical activity [20].

### Physical activity measurement

At the age four clinic visit, 665 children were fitted with an Actiheart monitor (Cambridge Neurotechnology Ltd, Papworth, UK) in order to measure their free-living physical activity. The Actiheart is a lightweight combined heart rate monitor and accelerometer, previously validated in preschool children [21,22]. The Actiheart unit was positioned in the midline, just below the xiphisternum and attached via a 70-100 mm wire to a smaller clip, horizontally to the left chest wall. Both parts were secured to the skin via standard electrocardiograph electrode pads. The Actiheart was set to record at 60-second epochs in order to maximise recording capacity and participants were asked to wear the monitor continuously for seven days, including when sleeping and during water-based activities. Monitors were returned by post, along with a maternal questionnaire to assess potential correlates of physical activity, previously validated in mothers of preschool children [23].

### Outcome variables

Only accelerometry data were used for this analysis, as equations to combine heart-rate and accelerometer data are yet to be developed for this age-group. Data were analysed using a bespoke program (MAHUffe [24]). All recordings between 11 pm and 6 am were removed with those between 9 pm and 11 pm removed if they included more than 45 minutes of sedentary time. This period was deemed to reflect the hours that children spent sleeping, and represents a conservative estimate of sleep time [25]. Data periods of 100 minutes or more with zero-activity counts were removed [26], as were days with <600 minutes of recording, with 10 hours of activity being the cut-off to define a valid day [27]. The accelerometer output was derived as counts per minute (cpm) for all children and thresholds for light (LPA, >20 cpm), moderate (>400 cpm), and vigorous (>600 cpm) activity were used to determine time spent at each activity intensity for each hour between 6 am and 11 pm. Moderate–to-vigorous activity (MVPA) was calculated as >400 cpm. True sedentary time was derived by subtracting ‘active’ time (>20 cpm) from total valid registered time. Average daily minutes spent at each activity intensity were also calculated for all children. The Actiheart cut-points, applying a conversion factor of 5, were derived and validated experimentally in children and adolescents [28,29]. These equate to thresholds of 100 counts for LPA and 2000 counts for MVPA in the Actigraph 7164 accelerometer (Actigraph, Pensacola, FL, USA), which are broadly aligned with the respective preschool-specific cut points [30-33].

### Exposure variables

Hour, time of day and week, and season (winter: December-February; spring: March-May; summer: June-August; autumn: September-November) were obtained from the accelerometer output. Each day was split into three periods: morning (6 am-12 pm), afternoon (12-5 pm) and evening (5-11 pm). These three time periods were chosen to reflect plausible segmentation of UK preschool-aged children’s days: formal preschool sessions usually begin or end at 12 pm, and children may remain in care until early evening (~5 pm). Child’s age, gender, and height assessed using a Leicester height measure, and weight assessed using calibrated digital scales (Seca, Ltd., Birmingham, UK) [20] were recorded during the visit. Height and weight were used to calculate child’s body mass index (BMI) (kg/m^2^) and child’s BMI z-score [34], indicating how many units of standard deviation a child’s BMI is above or below the average BMI value for their age and sex. BMI z-score was then used to categorise each child as under-weight, normal or over-weight/obese using the International Obesity Task Force classification [35]. Two variables were derived from the maternal questionnaire. Childcare, nursery or preschool attendance was classified as either full-time (≥30 hours per week), or part-time or other (<30 hours per week). The age that mothers left full-time education was used to derive three categories (≤16 years, 17-18 years and >18 years). Due to the study design, paternal data was not available.

### Statistical analysis

Analyses were carried out using STATA/SE 11 [36]. Demographic characteristics of children with (n = 593) and without valid physical activity data (n = 492) were compared using independent *t*-tests. A significance level of 0.05, set *a priori*, was used for all tests.

Descriptive characteristics of the sample and overall daily minutes spent in each activity intensity were calculated, along with the percentage contribution of each intensity to total activity.

To investigate the influences on children’s MVPA, LPA and sedentary activity, a series of two-level random intercept models were used. Daily observations at level 1 were nested within participants at level 2. For each outcome, average daily activity level and activity segmented across the day (morning, afternoon and evening) were assessed. Due to non-normality, the logarithm of MVPA was used for regression analyses. As these regression coefficients refer to the log-transformed outcome variable, beta values were exponentiated to give a ratio of the geometric means (GMR). A GMR can be interpreted similarly to a risk or odds ratio: any deviation from 1 indicates a% difference in MVPA relative to the respective reference category in the exposure variable. All models were adjusted for sex, weight status, age child’s mother left full-time education, time of the week (weekday vs. weekend) and season. Two sets of sensitivity analyses were conducted to explore the impact of including children with differing numbers of valid days: first including and excluding children with only one or two days of valid physical activity data (n = 49), and second children with and without activity data for both weekday and weekend days (n = 85).

## Results

A total of 593 children (89%) had valid physical activity data for one or more days (mean 5.1 (SD 1.4) days), with a mean wear time of just over 14 hours (mean: 852 (37.8) minutes) each day. We found no significant differences in child’s sex, maternal age at child’s birth or mother’s age leaving education between children who did and did not have valid activity data, suggesting included children are representative of the overall study population. Descriptive characteristics for the sample are presented in Table 1, with average daily activity levels and segmented across the day presented in Table 2.

**Table 1:** Characteristics of 4-year-old children with valid Actiheart data (n = 593).

Female (n (%))	300 (51)
Age (years)	4.1 (0.1)
Ethnicity (% non-white)	17 (3)
Children attend childcare full time	45 (8)
BMI z-score	0.15 (1.1)
Weight status (IOTF classification) (n (%))	
Underweight	42 (7)
Normal	474 (80)
Overweight/Obese	77 (13)
Age mother left education (n (%))	
≤16 years	190 (32)
17-18 years	213 (36)
≥18 years	190 (32)
Season of measurement (n (%))	
Winter	143 (24)
Spring	145 (24)
Summer	153 (26)
Autumn	155 (26)

All values are mean ± SD unless stated otherwise; SD: standard deviation; BMI: Body Mass Index; IOTF: International Obesity Task Force; Winter: December-February; Spring: March-May; Summer: June-August; Autumn: September-November.

**Table 2:** Children’s average daily activity levels and segmented across the day (in minutes)

	**Daily total (SD)**	**% Active time**^a^	**Morning**	**Afternoon**	**Evening**
Registered time	852.0 (37.8)	-	299.1 (44.9)	299.7 (4.3)	254.2 (34.6)
Sedentary	283.5 (72.2)	-	91.1 (40.0)	77.8 (46.6)	116.2 (44.2)
Active	568.5 (79.5)	-	208.0 (55.3)	221.9 (46.9)	138.0 (55.1)
Light	498.9 (65.8)	88%	185.4 (47.5)	189.9 (39.2)	123.6 (47.7)
MVPA	69.6 (30.7)	12%	22.6 (19.0)	32.0 (23.9)	14.4 (16.0)
Moderate	35.8 (14.9)	6%	11.7 (9.4)	16.0 (11.2)	8.0 (7.9)
Vigorous	32.8 (18.7)	6%	10.7 (11.6)	15.6 (15.2)	6.2 (9.7)

SD: Standard deviation; a: Percentage of average total daily active time that children spent at given intensity; MVPA: Moderate and vigorous physical activity;

Morning: 6 am-12 pm; Afternoon: 12-5 pm; Evening: 5-11 pm.

All children met the current UK recommended guidelines of 180 minutes of activity per day (at any intensity above sedentary) on 100% of days measured. On just over half of the days measured, children engaged in >60 minutes of MVPA (former activity guideline), with boys meeting this guideline on significantly more measurement days than girls (55 vs. 49% of measurement days, *p* < 0.001).

**Figure 1:**
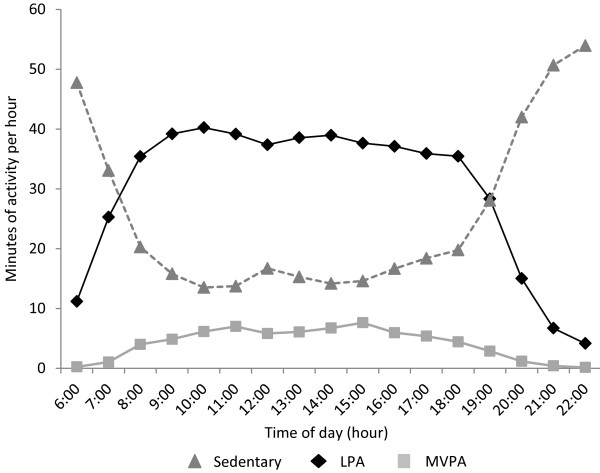
Four-year-old children’s average hourly activity patterns (n = 593).

Children’s average hourly activity levels across the day are shown in Figure 1. Although average daily time spent in sedentary activity, LPA and MVPA showed few differences by subgroups, multiple differential associations for all outcomes were seen when activity was segmented across the day (Tables 3, 4 and 5). Sensitivity analyses showed that results did not differ when children ≤2 days of valid activity data were included or excluded from analyses, nor when children with and without one valid weekday and weekend day were included or excluded. All children with ≥1 day of valid activity data were therefore included in analyses.

**Table 3:** Adjusted associations between daily sedentary time/segmented across the day and temporal and demographic factors

	**β**^**a **^**(95% C.I.)**			
	**Daily total**	**Morning**	**Afternoon**	**Evening**
Sex (ref: male)	10.7 (-1.9, 23.4)	6.3 (1.8, 10.8)**	2.8 (-3.1, 8.6)	2.9 (-2.4, 8.3)
Weight status (ref: normal weight)				
Overweight	12.3 (-7.0, 31.7)	1.6 (-5.3, 8.5)	3.7 (-5.3, 12.6)	6.7 (-1.5, 14.9)
Underweight	19.3 (-5.9, 44.5)	8.2 (-0.76, 17.3)	13.3 (1.6, 25.0)*	2.7 (-8.0, 13.4)
Child in full-time childcare (ref: part-time)	22.7 (-0.78, 46.0)	14.2 (5.9, 22.5)**	8.0 (-2.8, 8.7)	0.3 (-9.6, 10.2)
Age mother left full time education (ref: ≤16 years)				
17-18 years	0.75 (-14.7, 16.2)	-0.90 (-6.4, 4.6)	-1.2 (-8.2, 5.9)	3.1 (-3.5, 9.6)
≥18 years	13.7 (-2.1, 29.5)	3.0 (-2.6, 8.6)	0.40 (-6.8, 7.7)	10.8 (4.1, 17.4)**
Time of the week (ref: weekday)	-1.6 (-7.6, 4.3)	5.6 (2.9, 8.4)**	-3.7 (-6.6, -0.80)*	-2.8 (-5.8, 0.11)
Season (ref: winter)				
Spring	-3.6 (-20.9, 13.6)	0.39 (-5.5, 7.1)	-5.9 (-13.8, 2.0)	-1.2 (-8.5, 6.2)
Summer	-1.7 (-19.5, 16.0)	4.5 (-1.9, 11.0)	-0.10 (-8.3, 8.1)	-9.0 (-16.6, -1.4)*
Autumn	5.4 (-12.5, 23.3)	0.44 (-5.7, 6.6)	2.8 (-4.9, 10.6)	2.4 (-4.8, 9.6)

^*a*^β co-efficient represents the difference in minutes spent sedentary compared to the reference category for the given time segment. Final results from a two-level random intercept models adjusted for sex, weight status, time in childcare, age child’s mother left full-time education, time of the week (weekday vs. weekend) and season (Winter: December-February; Spring: March-May; Summer: June-August; Autumn: September-November); *p < 0.05; ** *p* < 0.01.

**Table 4:** Adjusted associations between daily LPA/segmented across the day and temporal and demographic factors

	**β**^**a **^**(95% C.I.)**			
	** Daily total**	** Morning**	** Afternoon**	** Evening**
Sex (ref: male)	-11.1 (-22.7, 0.47)	-8.5 (-14.6, -2.4)**	-0.70 (-5.4, 4.0)	-2.9 (-9.0, 3.2)
Weight status (ref: normal weight)				
Overweight	3.4 (-14.3, 21.3)	5.6 (-3.7, 15.1)	-0.26 (-7.5, 7.0)	-2.6 (-12.0, 6.7)
Underweight	-4.2 (-27.3, 18.9)	-6.0 (-18.3, 6.2)	-7.6 (-17.1, 1.8)	3.4 (-8.9, 15.6)
Child in full-time childcare (ref: part-time)	-11.7 (-33.2, 9.8)	-12.4 (-23.8, -1.1)*	-4.2 (-12.9, 4.6)	6.1 (-5.2, 17.4)
Age mother left full time education (ref: ≤16 years)				
17-18 years	-1.4 (-15.6, 12.8)	3.2 (-4.3, 10.7)	-0.40 (-6.2, 5.4)	-5.1 (-12.6, 2.4)
≥18 years	-13.9 (-28.4, 0.6)	6.2 (-1.4, 13.8)	-2.9 (-8.7, 3.0)	-15.3 (-22.9, -7.7)**
Time of the week (ref: weekday)	-3.7 (-9.1, 1.6)	-11.3 (-14.0, -8.6)**	0.17 (-2.5, 2.8)	7.9 (5.0, 10.8)**
Season (ref: winter)				
Spring	2.6 (-13.1, 18.3)	6.3 (-1.9, 14.4)	1.5 (-4.9, 8.1)	-5.2 (-13.4, 3.1)
Summer	-0.44 (-16.7, 15.8)	-10.1 (-18.6, -1.5)*	-1.9 (-8.6, 4.9)	7.7 (-0.95, 16.3)
Autumn	-7.6 (-24.0, 8.8)	-1.2 (-9.2, 6.7)	-3.1 (9.5, 3.3)	-6.0 (-14.0, 2.1)

^*a*^β co-efficient represents the difference in minutes spent in light physical activity (LPA) compared to the reference category for the given time segment. Final results from a two-level random intercept models adjusted for sex, weight status, time in childcare, age child’s mother left full-time education, time of the week (weekday vs. weekend) and season (Winter: December-February; Spring: March-May; Summer: June-August; Autumn: September-November); *p < 0.05; ** *p* < 0.01.

**Table 5:** Adjusted associations between daily MVPA/segmented across the day and temporal and demographic factors

	**Exponentiated β**^**b **^**(95% C.I.)**			
	**Daily total**	**Morning**	**Afternoon**	**Evening**
Sex (ref: male)	0.85 (0.77, 0.94)**	0.82 (0.73, 0.91)**	0.93 (0.84, 1.04)	0.83 (0.74, 0.94)**
Weight status (ref: normal weight)				
Overweight	0.93 (0.80, 1.09)	0.91 (0.76, 1.08)	0.92 (0.79, 1.09)	0.94 (0.78, 1.13)
Underweight	0.86 (0.70, 1.04)	0.89 (0.70, 1.12)	0.84 (0.68, 1.03)	1.01 (0.79, 1.28)
Child in full-time childcare (ref: part-time)	0.79 (0.66, 0.94)**	0.80 (0.65, 0.99)*	0.88 (0.72, 1.01)	1.04 (0.83, 1.30)
Age mother left full time education (ref: ≤16 years)				
17-18 years	0.96 (0.85, 1.06)	0.97 (0.85, 1.12)	1.01 (0.89, 1.15)	0.84 (0.73, 0.98)*
≥18 years	0.94 (0.83, 1.06)	0.98 (0.85, 1.13)	1.05 (0.92, 1.19)	0.72 (0.62, 0.84)**
Time of the week (ref: weekday)	0.97 (0.93, 1.02)	0.83 (0.78, 0.89)**	1.09 (1.03, 1.17)**	1.08 (1.00, 1.17)*
Season (ref: winter)				
Spring	1.18 (1.03, 1.36)*	1.08 (0.92, 1.27)	1.07 (0.93, 1.24)	1.11 (0.94, 1.31)
Summer	1.15 (1.01, 1.33)*	0.93 (0.79, 1.09)	1.07 (0.92, 1.24)	1.38 (1.16, 1.64)*
Autumn	1.08 (0.93, 1.23)	0.99 (0.85, 1.16)	0.98 (0.85, 1.12)	0.98 (0.83, 1.15)

^b^Exponentiated β or geometric mean ratio (GMR) - any deviation from 1 indicates a% difference in moderate and vigorous physical activity (MVPA) relative to the reference category for the given time segment. Final results from a two-level random intercept models adjusted for sex, weight status, time in childcare, age child’s mother left full-time education, time of the week (weekday vs. weekend) and season (Winter: December-February; Spring: March-May; Summer: June-August; Autumn: September-November); *p < 0.05; ** *p* < 0.01.

Girls were more sedentary (β = 6.3, (95% C.I. 1.8, 10.8)) and less active (LPA: -8.5 (-14.6, -2.4)) in the mornings, engaging in 18% less MVPA (GMR:0.82 (0.73, 0.91)) compared to boys. Girls also engaged in less MVPA in the evenings (0.83 (0.74, 0.94)). Underweight children were more sedentary in the afternoons compared to their normal weight peers. Compared with children whose mothers left school at or before 16, children whose mothers left school after 18 were more sedentary and less active (engaging in less LPA and MVPA) in the evenings.

Children who attended preschool full-time were more sedentary and less active in the mornings compared to children attending part-time. Moreover, children were also more sedentary and less active on weekend mornings compared to weekdays. The reverse was true on weekend afternoons and evenings: children were less sedentary and engaged in more MVPA on weekend afternoons, and were more active in the evenings compared to weekdays. Compared to winter months, children engaged in less LPA in the morning during summer, but were also less sedentary and engaged in more MVPA on summer evenings.

## Discussion

This study showed that British 4-year old children spend on average 67% of their day being active, engaging predominately in LPA (~88% of active time). Although few differences in average activity levels were observed, the current study provides novel information about how temporal and demographic factors differentially influence children’s activity when segmented across the day. Child’s sex and weight status, age their mother left full-time education, attending childcare full-time, time of the week and season were all independently associated with children’s activity levels at different times of the day. These time-specific observations are likely to be important for intervention development, indicating that focussing on (specific subgroups in) periods when children are less active, for example girls in the mornings, or during winter months, may result in larger increases in children’s activity.

Based on the data presented here, all children met the current UK activity guidelines. Although these guidelines are comparable to Australian recommendations, previous estimates indicate that only 5.1% of Australian children met these guidelines [17]. The proportion of time children spent active in this study (~67%) was also higher than reported previously in studies in Europe (~16%) [37,38] and Australia(~16%) [17]. Several factors, including the population studied, the measure of physical activity and wear and processing protocols, are likely to contribute to these differences across studies. These data were collected in a population-based sample, as opposed to a pre-school or care-setting based sample as is more commonly used [17,38]. Moreover, previous studies have predominantly used Actigraph accelerometers worn on the hip during waking hours and taken off during water-based activities [17,37,38]. The waterproof Actiheart monitor, worn continuously for 24 hours each day, is therefore likely to have captured more of children’s daily activity. Further, the cut points used here, equivalent to those previous applied to Actigraph data [30,31,39], were derived experimentally and validated against doubly labelled water [29]. As other studies have used higher cut points to classify active time [17,37,38], they are likely to report lower levels of activity. Taken together, varying study methods and lack of raw count per minute data to compare between studies [40], make comparison of prevalence estimates of young children’s activity, and their compliance with guidelines, problematic [27].

We observed that children spend the majority of their time in LPA. Provided that ≥100 cpm is a valid threshold for LPA [30,31], and even accounting for differences in classification according to accelerometer cutpoints [41], children’s light activity levels are ‘sufficient’ to comfortably satisfy current activity guidelines. It may however be important to consider whether specifying activity intensity along with a total activity guideline is necessary for this age-group. LPA may confer limited health benefits for young children, if, as is the case in older children and adults, benefits to health are associated with more vigorous intensity activity [42,43]. Indeed, in this sample of children, MVPA (and not LPA) has been found to be positively associated with bone density [44] and vigorous activity with lower fat mass [45]. The relevance of this debate is further highlighted by the recent observation that more than 1 in 5 UK children are either overweight or obese on entering school at age 5 [46], which seems at odds with observations that all children are sufficiently active. The importance of activity intensity in younger children therefore requires further investigation to inform future activity guidelines for the under-5 s.

Increasingly, evidence suggests that even at a young age boys are more active than girls [17,37,47], which was also observed here. This study adds to the current literature by highlighting that these differences are not consistent throughout the day. Sex differences were most apparent in the mornings, with girls being relatively less active and more sedentary than boys. In contrast, children’s weight status did not appear to have an influence on children’s activity across the day. As similar finding have been seen previously in this age group [18], these patterns may be indicative of preschool-aged children’s activity across the day, and future interventions may need to consider a temporal focus to differentially target boys and girls.

Interestingly, children who attended nursery full-time compared to part-time were more sedentary and less active in the mornings. It has been suggested previously that childcare may influence children’s physical activity levels [10], but the measure used here provides only a basic idea of whether children were usually in childcare full- or part-time. As it was not possible to determine where children were during the measurement week, further work is required to determine whether activity levels differ by time spent in childcare and what factors in the childcare environment influence children’s activity.

The differences in daily patterns observed between week and weekend days are likely to reflect families’ normal working weeks, with children typically rising and going to bed earlier on weekdays. What is not clear is why children whose mothers left school at a later age were also more likely to be sedentary and less active in the evenings. As maternal employment status was not collected at the age four visit, it is not possible to determine whether this finding relates to more of these mothers being in full-time work. However, it has been shown previously that three-year-old children with more highly educated mothers are more likely to have a set bedtime and bedtime routine [48], which may plausibly account for the children of higher educated mothers being less active in the evenings here.

Although not studied directly, it is likely that the observed seasonal variation, with greater activity and less sedentary time in the evenings during the summer compared to winter, reflects the longer daylight hours of summer months. Contrary to previous observations in preschool children [49], children’s average daily MVPA was higher in spring compared to winter, which appears to be the result of a consistent accumulation of more activity throughout the day. Through this novel and detailed investigation of the temporal differences in behaviour, we are able to highlight opportunities to encourage activity in children, such as specifically targetting activity during the mornings for girls, or facilitating day-time activity opportunities for preschoolers during the autumn and winter months.

### Strengths and limitations

Using data from a large population-based sample of four-year-old children, this study is one of the first to describe objectively measured physical activity levels of British preschool-aged children [50,51], and highlight differences in activity across the day. As mothers were recruited before children were born, children were drawn from all socio-economic strata in the city of Southampton and surrounding areas. The sample is not therefore subject to biases seen in more commonly used (pre-)school-based samples, which by their nature exclude children who do not attend formal childcare (for sufficient amounts of time). In addition, children’s activity was measured throughout the year, rather than being bounded by school terms, allowing analysis of seasonal variation.

We included all children with at least one valid day of physical activity data to maximise our sample size and power. However, sensitivity analyses showed that restricting the sample to those with valid physical activity for ≥3 days or to those with at least one weekday and weekend day did not alter the results. We also found no significant differences between those who did and did not provide valid activity data at age 4, suggesting that the sample is likely to be representative of the Southampton study population and wider population. This said, participants were predominately white British in keeping with the Southampton region (~82%) [52], and fewer children in this sample were overweight or obese compared to the national average [46]. Care should therefore be taken in generalizing these results to specific sub-populations, including those from ethnic minorities or populations with high levels of childhood overweight/obesity.

The novel use of time-stamped data, dividing days into three time periods, allows a more detailed description of the temporal patterns of children’s activity across the day. These segments reflect plausible subdivision of preschool-aged children’s days, with sessional care offered from Monday-Friday in the UK. Although most children in this sample attended childcare at least part-time, time-matched care attendance data was not available to determine what influence childcare attendance had on their physical activity levels. However, by segmenting the day in this way, clear times do appear during which public health interventions to increase activity in specific subgroups may be more likely to show a beneficial impact.

Actiheart monitors present a valid [21,22] and feasible form of measuring young children’s activity, providing increased wear time and therefore enhanced characterization of children’s daily activity levels, in combination with a validated questionnaire [23]. Validation studies have shown that the use of 60-second epochs, used here to allow sufficient memory to record for 7 days, may underestimate MVPA [53], whilst overestimating LPA [54]. Whilst this may have led to attenuation of the associations found here, our results still suggest that factors influencing children’s sedentary, LPA and MVPA are likely to differ in the preschool-aged population.

## Conclusion

Four-year old British children accumulate sufficient activity over the day to meet current guidelines, although this activity is predominantly of light intensity for which the health benefits remain to be determined. In addition, patterning of activity varies over the day by demographic and temporal factors. These should be taken into account when developing new interventions to increase preschoolers’ physical activity, for example focusing on encouraging girls’ morning activity and providing opportunities for daytime activity in winter months.

## Abbreviations

PA: Physical activity; LPA: Light physical activity; MVPA: Moderate and vigorous physical activity; SWS: Southampton Women’s Survey; cpm: Counts per minute; BMI: Body mass index; GMR: Geometric mean ratio.

## Competing interests

The authors declare that they have no competing interests.

## Authors’ contributions

KRH was responsible for cleaning and analysing the physical activity data, conducting the data analyses and interpretation of the results, drafting the manuscript. AMM contributed to data analyses and interpretation, SJS provided statistical assistance and PJC assisted in cleaning the physical activity data. NCH, HMI, KMG & CC were responsible for the overall SWS study concept, design and oversaw the collection of data UE & EvS were responsible for conceptualisation and designed of physical activity data collection, designed the data collection instruments. EvS additionally provided input on the data analyses and interpretation of the results. All authors critically reviewed the manuscript and approved its final version.
